# White blood cell inflammatory markers are associated with depressive symptoms in a longitudinal study of urban adults

**DOI:** 10.1038/tp.2016.180

**Published:** 2016-09-20

**Authors:** M A Beydoun, H A Beydoun, G A Dore, J-A Canas, M T Fanelli-Kuczmarski, M K Evans, A B Zonderman

**Affiliations:** 1 Laboratory of Epidemiology and Population Sciences, NIH Biomedical Research Center, National Institute on Aging, IRP, Baltimore, MD, USA; 2 Department of Medicine, Johns Hopkins Medical Institutions, Baltimore, MD, USA; 3 Pediatric Endocrinology, Diabetes and Metabolism, Nemour's Children's Clinic, Jacksonville, FL, USA; 4 Department of Behavioral Health and Nutrition, University of Delaware, Newark, DE, USA

## Abstract

Total white blood cell count (TWBCC) and percentage (%) composition of lymphocytes (PL) or neutrophils (PN) are linked to mid- and late-life depression, though sex-specific temporal relationships between those inflammatory markers and depressive symptoms remain unclear. The association between inflammation and depressive symptoms in longitudinal data on ethnically and socioeconomically diverse urban adults was examined with two hypotheses. In hypothesis 1, we examined the relationship between TWBCC, PL and PN with change in level of depressive symptoms from baseline to follow-up, stratifying by sex. In hypothesis 2, we examined reverse causality, by testing the relationship of depressive symptoms with change in TWBCC, PL and PN. Multiple linear mixed-effects regression models were performed to examine both the hypotheses. The sample sizes of participants (*n*) and repeated observations (*n*') were: Hypothesis 1 (*n*=2009; *n*'=3501); Hypothesis 2 (*n*=2081; *n*'=3560). Among key findings (Hypothesis 1), in women, higher TWBCC was linked to a faster increase in depressive symptom total score (γ_1112_±s.e.: +0.81±0.28, *P*=0.003), with a slower increase over time in the positive affect subdomain coupled with faster increases in depressed affect and somatic complaints. Among women, baseline score on somatic complaints was positively associated with low PN (γ_01a_=+1.61±0.48, *P*<0.001) and high PL (γ_01a_=+1.16±0.45, *P*=0.011), whereas baseline score on positive affect was inversely related to higher PL (γ_01a_=−0.69±0.28, *P*=0.017). Results among men indicated that there was a positive cross-sectional relationship between low TWBCC and depressive symptoms, depressed affect and an inverse cross-sectional relationship with positive affect. However, over time, a low TWBCC in men was linked to a higher score on positive affect. There was no evidence of a bi-directional relationship between WBC parameters and depressive symptoms (Hypothesis 2). In sum, TWBCC and related markers were linked to depressive symptoms, mostly among women. Further longitudinal studies are needed to replicate this sex-specific association.

## Introduction

Unipolar depression is a chronic condition^1, 2^ accounting for a major part of the health care system,^3^ with a lifetime prevalence among American adults of ~12% in men and ~21% in women.^3^ Depression and elevated depressive symptoms in general were linked to altered immunity.^4, 5, 6, 7, 8, 9, 10, 11, 12, 13, 14, 15, 16, 17, 18, 19, 20, 21, 22, 23, 24, 25, 26, 27, 28, 29, 30, 31, 32, 33, 34, 35, 36, 37^ Several studies included in meta-analyses found an association of elevated depressive symptoms with WBC-related markers of inflammation including increased absolute leukocyte levels (that is, total white blood cell count (TWBCC)), reduced percentage lymphocytes (PL) out of total leukocytes or lymphopenia, and increased percentage neutrophils (PN) or neutrophilia. However, the results of these meta-analyses^38, 39, 40^ had distinctive conclusions mainly owing to heterogeneity in study design, depressive symptoms measures, potential confounders considered, measured inflammatory markers and sample characteristics (for example, age, sex, ethnic background and health behaviors including diet). Nevertheless, as reverse causality cannot be ruled out, bi-directional associations between white blood cell (WBC)-related markers (TWBCC, PN and PL) and depressive symptoms in populations are under-studied, particularly in terms of sex-specific longitudinal changes in one as predicted by the baseline value of the other.^24, 28^

Using longitudinal data on a large and ethnically diverse urban sample, our study had three separate objectives and two hypotheses. The first objective (Objective 1) was to assess the cross-sectional (that is, baseline vs baseline) and bi-directional longitudinal (that is, baseline vs rate of change) relationships of WBC-related markers (that is, high/low TWBCC, high/low PN, high/low PL; high: >90th percentile, low: <10th percentile) with depressive symptoms, while the second objective (Objective 2) was to assess the cross-sectional and longitudinal relationships of WBC-related markers with specific domain of depressive symptomatology. Finally, the third objective (Objective 3) was to examine whether the association in the above two objectives are sex-specific. Two longitudinal relationships were tested: Hypothesis 1: baseline WBC-related markers predict rate of change in depressive symptoms and Hypothesis 2: baseline depressive symptoms predict WBC-related markers.

## Materials and methods

### Database and study sample

The healthy aging in neighborhoods of diversity across the lifespan (HANDLS) study is an ongoing prospective cohort study initiated in 2004 that recruited a representative sample of African Americans and whites aged 30–64 years old and living in Baltimore, MD, USA.^41^ The HANDLS design consisted of an area probability sample of 13 neighborhoods (groups of contiguous census tracts), with a first phase at baseline that screened and recruited participants and administered a household interview. At phase II, in-depth examinations in mobile medical research vehicles were conducted. Written informed consent was obtained after access to a protocol booklet in layman's terms along with a video describing all the procedures and future re-contacts. The MedStar Institutional Review Board approved all the materials. Longitudinal data from baseline (visit 1, also known as wave 1, ended in 2009) and the first follow-up examination (visit 2, also known as wave 3, ended in 2013, mean±s.d. follow-up=4.65±0.93 years) were used in this study.

Initially, 3720 participants were recruited (Sample 1), Center for Epidemiologic Studies-Depression (CES-D) data were available for *N*=2725 participants at baseline and *N*=2258 at wave 3; while wave 1 TWBCC, PN and PL were available for *N*=2744–2745 participants and wave 3 TWBCC, PN and PL were available for *N*=2254–2266. We restricted our sample to those with 2 days of dietary recall at wave 1 and CES-D data at either wave, while further limiting the analyses to those with baseline TWBCC, PN and PL for Hypothesis 1, along with non-missing covariates (*n*=2009–2011; Sample 2A, Hypothesis 1; repeated observations *n*=3496). Participants in Sample 2A compared with remaining HANDLS participants in Sample 1 had a lower percentage with poverty income ratio >125% (56.4% vs 62.1%, *P*=0.005), and a higher percentage of women (56.5% vs 52.0%, *P*=0.009) with no significant differences by age and race distributions.

In addition, for Hypothesis 2, the final sample size consisted of participants with complete WBC variables at either wave and complete data on CES-D total score at wave 1 (Sample 2B, *n*=2081; 908 men and 1173 women; repeated observations: *n*'=3560), with similar selectivity patterns as for Sample 2A.

### Depressive symptoms

Depressive symptoms were operationalized using the CES-D, at both baseline and follow-up. The 20-item CES-D is a self-reported symptom rating scale assessing affective and depressed mood.^42^ A score of ⩾16 on the CES-D is reflective of elevated depressive symptoms (EDS),^43^ and predicts clinical depression based on the Diagnostic and Statistical Manual, fourth edition (DSM-IV) criteria.^44^ Four CES-D sub-domains exhibiting an invariant factor structure between The National Health and Nutrition Examination Survey I and pilot HANDLS data^45^ were computed. We tested our hypotheses using total and domain-specific CES-D scores: (1) somatic complaints; (2) depressive affect; (3) positive affect and (4) interpersonal problems.^45^

### White blood cell inflammatory markers

Fasting blood samples were collected from participants at baseline and follow-up to determine TWBCC, (K mm^−3^) and percentage WBC subtypes, including PN and PL, using electronic cell sizing, counting, cytometry and microscopy (http://www.questdiagnostics.com/testcenter/TestDetail.action?ntc=7064). Blood samples were transported to Quest diagnostics for analysis in which technicians were blinded to other study parameters, including depressive symptoms levels.

### Covariates

#### Socio-demographic, lifestyle and health-related potential confounders

Potential confounders in our analytic models included age, sex, race (white vs African American), educational attainment (0⩽high school (HS); 1=HS and 2⩾HS), poverty status (below vs above 125% the federal poverty line), measured body mass index (BMI; kg/m^2^), current use of drugs ('opiates, marijuana or cocaine'=1 vs not=0), and current smoking status (0: 'never or former smoker' and 1 'current smoker').

#### Dietary potential confounders

We entered dietary potential confounders that were linked to depression based on previous studies and included vitamins B-6, folate and B-12, total carotenoids (α-carotene, β-carotene, β-cryptoxanthin, lutein+zeaxanthin, lycopene), vitamin C and α-tocopherol^46, 47, 48, 49, 50, 51, 52, 53, 54^ (all divided by total energy intake and expressed per 1000 kcal) and the ratio of n-3 polyunsaturated fatty acid (PUFA):n-6 PUFA.^55^ Total energy intake was entered as a covariate to emulate a multivariate nutrient density model.^56^ Models also included overall dietary quality as measured by the Healthy Eating Index (HEI-2010) total score, with details provided by the National Cancer Institute's Applied Research (http://appliedresearch.cancer.gov/tools/hei/tools.html) and the HANDLS (http://handls.nih.gov/06Coll-dataDoc.htm) websites.

### Statistical analysis

Stata 14.0 (StataCorp, College Station, TX, USA) was used in all the analyses.^57^ First, baseline characteristics were examined for men vs women, further differentiated by EDS status (CES-D score ⩾16 vs <16, based on mean score across waves), using *t*-tests and analysis of variance for continuous variables and *χ*^2^ tests for categorical variables. Second, several mixed-effects regression models on continuous CES-D total or on domain-specific score(s) were conducted to test associations with three alternative WBC exposures, controlling for potential confounders. Sex-specific associations were tested by adding interaction terms to the multivariable mixed-effects regressions and stratifying by sex. Supplementary Appendix I outlines methodology used to run mixed-effects regression models, which were previously conducted.^58, 59, 60^

Adjustment for non-random participant selection bias was done with a two-stage Heckman selection process.^60, 61, 62^ An *α*=0.05 was used for all the analyses, and *P*-values >0.05 and <0.10 were considered borderline significant for main effects, whereas a *P*-value <0.10 was considered significant for interaction terms^63^ before multiple testing correction. The process of correcting for multiple testing was done using the family-wise Bonferroni correction,^64^ in which we assume that each of total CES-D and sub-domains of CES-D are distinctive outcomes for which we test the association with the three WBC-related exposures that conceptually related. A similar approach was used in other previous studies.^60, 65^ Accounting for three exposures, type I error was reduced to 0.05/3=0.017 for main effects and 0.10/3=0.033 for interaction terms.

## Results

Across-visit EDS^+^ was more prevalent among women vs men (46.6% vs 36.5%, *P*<0.001, *χ*^2^ test; Table 1). EDS^+^ participants had a lower prevalence of poverty income ratio ⩾125%, a higher likelihood of unemployment and lower educational attainment, a higher proportion of current smokers overall and current illicit drug users (in women), compared with EDS^−^ participants. Despite women having a higher mean BMI compared with men (31.3 vs 28.0), BMI was not linked to EDS status. There were also consistently lower prevalence rates of current smoking and drug use among women compared with men. Poorer dietary quality (HEI-2010) was observed among men vs women, and among EDS^+^ (vs EDS^−^) participants, for both sexes. Mean micronutrient intakes per 1000 kcal at baseline were lower among EDS^+^ vs EDS^−^, specifically vitamin E, vitamin B-6, folate and vitamin C (women). In terms of WBC exposures, TWBCC was higher among EDS^+^ vs EDS^−^ women (*P*=0.02), with women overall having higher TWBCC vs men (*P*=0.03). PN was higher in women who were also more likely to be in the <10th percentile of the distribution (9.9% vs 5.9%, *P*=0.002), when EDS^+^ vs EDS^−^. Similarly, EDS^+^ vs EDS^−^ women were more likely to be above the 90th percentile of PL.

**Figure 1:**
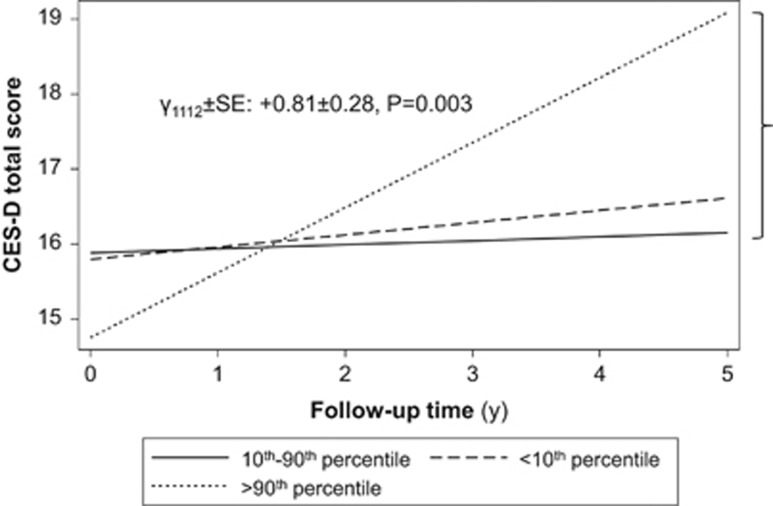
Predictive margins of CES-D total score by total white blood cell count (TWBCC) among women from multiple linear mixed-effects regression model, HANDLS 2003–2013. CES-D, Center for Epidemiologic Studies-Depression; HANDLS, healthy aging in neighborhoods of diversity across the lifespan.

For Hypothesis 1 and Objective 1, we first tested cross-sectional and longitudinal relationships between WBC-related markers and depressive symptoms. This was examined with mixed-effects linear regression models (Supplementary Appendix I), with results presented in Table 2. The TWBCC main effect parameter reflects the cross-sectional association of TWBCC with depressive symptoms, whereas TWBCC × time interaction can be interpreted as the effect of TWBCC on the annual rate of change in depressive symptoms. Two exposure levels were compared with the 10th–90th percentile range, namely 'Low' (<10th percentile) and 'High' (>10th percentile). We found that for men there was a cross-sectional association between the <10 percentile of TWBCC and depressive symptoms (γ_0111_=+2.51±1.05, *P*=0.016) but no cross-sectional association between PN or PL and depressive symptoms, nor was there an association for any of the WBC-related markers and depressive symptoms longitudinally. For woman, high TWBCC was linked to an accelerated increase in CES-D score over time (γ_1112_=+0.81±0.28, *P*=0.003; Figure 1). In a separate model, also among women, baseline PN<10th percentile (<44.1%) was associated with higher baseline CES-D total scores compared with 10th–90th percentile range, adjusting for multiple covariates (γ_0121_=+2.70±1.26, *P*=0.031). This result, however, did not survive multiple testing.

Pertaining to our second objective, we examined whether WBC-related markers are associated with specific domains of depressive symptomatology. To this end, Table 3 (Hypothesis 1, Objective 2) shows a series of comparable linear mixed-effect regression models for women, adjusting baseline CES-D subdomain scores and rate of change over time for the same covariates and entering each WBC exposure into a separate model (Supplementary Appendix I). After adjustment for multiple testing (*α*: 0.10 → 0.033), high TWBCC (>90th vs 10th–90th percentile) was associated with faster increase in somatic complaints and depressed affect and a slower increase in positive affect (higher positive affect → less depressive symptoms). Baseline scores on somatic complaints were also positively associated to low PN (γ_01a_=+1.61±0.48, *P*<0.001) and high PL (γ_01a_=+1.16±0.45, *P*=0.011) among women; whereas baseline score on positive affect was inversely related to higher PL (γ_01a_=−0.69±0.28, *P*=0.017).

Table 4 (Hypothesis 1, Objective 2) shows results among men for associations between WBC-related markers and CES-D component scores at baseline and rates of change over time. After adjustment for multiple testing, several key findings emerged. First, higher baseline TWBCC was linked to higher depressed affect and lower positive affect. Nevertheless, the longitudinal effects indicated that low TWBCC was associated with a slower increase in depressed affect and faster increase in positive affect. PL and PN were not associated with cross-sectional or longitudinal change in CES-D sub-domains among men.

Objective 3 of Hypothesis 1 was to examine whether cross-sectional and longitudinal associations between WBC exposures and depressive symptoms (total score and domains) differed significantly between men and women. This was done by adding relevant interaction terms in the mixed-effects regression models. First, the effect of PN<10th percentile (<44.1%) on baseline CES-D total score compared with 10th–90th percentile differed significantly between men and women. Second, the cross-sectional association between somatic complaints and low PN (γ_01a_=+1.61±0.48, *P*<0.001) as well as high PL (γ_01a_=+1.16±0.45, *P*=0.011) among women was found to be significantly different between men and women.

Finally, we examined all three objectives pertaining to Hypothesis 2 to test sex-specific associations between baseline depressive symptoms and rate of change in WBC-related markers (Supplementary Table S1), using a similar series of multiple mixed-effects regression models, stratifying by sex. Baseline CES-D total score was not associated with any of the WBC-related outcomes. The same finding pertained to depressive symptom domains (data not shown) and the associations did not differ between men and women.

## Discussion

This study is among few to examine bi-directional relationships between WBC-related inflammatory markers and depressive symptoms, and we believe is the first to examine those relationships differentially between men and women in an urban population of adults. Higher TWBCC was linked to a faster increase in depressive symptoms among women, with a slower increase over time in the positive affect sub-domain, coupled with faster increases in depressed affect and somatic complaints. Results among men indicated that there was a positive cross-sectional relationship between low TWBCC and depressive symptoms, depressed affect and an inverse cross-sectional relationship with positive affect. However, over time, a low TWBCC in men was linked to a higher score on positive affect (See Supplementary Table S2 for summary of key findings).

TWBCC is an under-studied inflammatory marker of depression, with studies limited to small case–control design,^6, 16, 31^ and a handful of cross-sectional and longitudinal studies.^21, 24, 34, 35, 36^ Bi-directional relationships were examined only in one study.^24^ In the largest study to date (*N*=11 367–11 857, EPIC-Norfolk, age:40–80 years), after multivariate adjustment for age and cigarette smoking, an association between major depressive disorder and TWBCC count was reported in men, but not women.^34^ Our study shows that the longitudinal association between TWBCC and depressive symptoms and sub-domains was restricted to women and in the expected direction (that is, higher TWBCC increases the rate of depression over time). In contrast, significant findings among men were mostly cross-sectional indicating that lower TWBCC may be associated with elevated depressive symptoms at baseline, with the possible exception of positive affect rate of change, which was directly linked to lower TWBCC among men.

In another large cross-sectional study (*n*=3769 participants (EPESE)), TWBCC count was higher among EDS^+^ compared with the EDS^−^ group,^21^ though results were not sex-specific. This result was replicated in a cohort study of cardiovascular disease-free adults (453 men (19–89 years old) and 400 women (18–84 years old)), in which the Zung Self-Rating Depression Scale (range 0–100) total score was positively correlated with TWBCC, after multivariate adjustment.^35^ Baseline depressive symptoms independently predicted higher subsequent TWBCC, but not vice versa, which is at odds with our results.^35^ Our study design differed from the latter's by including both healthy and CHD participants and participants with other comorbidities and selecting both whites and African American adults.^35^

In our study, among women, baseline somatic complaint score was positively associated with low PN and high PL, whereas baseline positive affect score was inversely related to higher PL. Similarly, WBC subtypes (including PL and PN) or WBC activity (for example, natural killer cell activity) in relation to depressive outcomes were examined in mostly small case–control studies.^4, 5, 6, 7, 8, 9, 10, 11, 12, 13, 14, 17, 18, 20, 30, 31, 37^ Notwithstanding these controversial findings, a recent meta-analysis indicates that depressed individuals tend to have a state of neutrophilia (that is, high PN) coupled with lymphopenia (that is, low PL).^40^ In studies of depression with a higher percentage of females, depressed participants have greater proportional increases in B- and T-cell numbers compared with the non-depressed, indicating lymphocytosis predominates.^40^ These findings agree with our results for female participants of HANDLS. A study by Nakata *et al.*^28^ investigated the putative temporal bi-directionality between depressive symptoms and counts of lymphocyte subsets and found that depressive symptoms preceded variation in follow-up lymphocyte counts, particularly NK cells. In contrast, baseline NK cells were not predictive of follow-up depressive symptoms.^28^ Our study indicates that baseline depressive symptoms are associated with baseline PL and PN, specifically among women, without any indication of baseline depressive symptoms affecting or being affected by those parameters over time.

Biological mechanisms behind baseline leukocytosis and increase in depressive symptoms among women are largely speculative. One potential mechanism is chronic stress stimulating hematopoietic stem cells in the bone marrow, increasing blood cortisol (hypercortisolemia) and reducing vagal nerve activity, possibly leading to the accelerated production of WBCs.^66, 67^ Our findings, however, indicate that although TWBCC can result from chronic stress, depressive symptoms among women worsened over time with pre-existing elevated TWBCC. In contrast, elevated baseline depressive symptoms did not alter the rate of change in TWBCC in both sexes. Another possibility is that chronic stress could lead to poor health behaviors, including smoking, sedentary behavior and poor diet, which could lead to central adiposity and inflammation, independent of BMI.^68, 69^ Those factors themselves could influence immunity by increasing levels of inflammation, partly manifested by a higher TWBCC. Consequently, TWBCC becomes an adverse exposure increasing depressive symptoms over time, or reducing the rate of increase in positive affect. Many of those adverse health behaviors, excluding physical activity and central adiposity, were adjusted for in this model. It is possible that other mediators may have a role, including poor sleep which was found to mediate this relationship in at least one other study.^24^ Residual confounding by those factors included cannot be ruled out, given that those measures were self-reported and liable to measurement error.^24^ Moreover, when injected in non-depressed individuals, monokines, specifically interleukin-1, can trigger symptoms suggestive of depression based on the DSM-III-R by provoking hormonal changes.^70^ Estrogen can activate macrophages and produce monokines, which explains the 3:1 female/male incidence of depression ratio in many adult populations.^70^ Our findings indicated that higher TWBCC in females can lead to a faster increase in depressive symptoms, potentially due to a synergistic effect between estrogen and stress hormones in the presence of chronic stress.

Our study has several notable strengths. First, being longitudinal in design, this study unveiled temporality of associations between WBC-related markers and depressive symptoms. The relationship was in the direction of baseline WBC markers→ depressive symptoms but not vice versa. Second, the analytic sample size permitted stratification by sex without significant loss in statistical power. Third, a large number of covariates were entered into the mixed-effects regression models to control for confounding effects at the level of the intercept as well as the slope. Nevertheless, our study findings should be interpreted with caution in light of several limitations, including potential selection bias owing to non-participation, which was minimized using a two-stage Heckman selection model. Moreover, despite the direct measures of WBC markers, other variables including the CES-D were self-reported, leading to measurement error and residual confounding in the main associations of interest.

In sum, WBC-related markers were linked to depressive symptoms, mostly among women. Further longitudinal studies are needed to replicate this sex-specific association. In addition, adding a third wave of data would allow examining potential mediating factors between those markers of inflammation and depressive symptoms, particularly TWBCC. Finally, studies are needed to distinguish between behavioral and genetic factors behind those associations.

## Figures and Tables

**Table 1:** Characteristics of HANDLS study participants by sex and CES-D score (mean across waves)^aa^ Values are percent or Mean±s.e.m.

	*Men*			*Women*			P^bb^ *P*-value was based on independent samples *t*-test when row variable is continuous and *χ*^2^ test when row variable is categorical.		
	*<16 (*n=*556)*	⩾*16 (*n=*319)*	*All men (*n=*875)*	*<16 (*n=*606)*	⩾*16 (*n=*528)*	*All women (*n=*1134)*	*Men* vs *women*	*Low* vs *high CES-D score among men*	*Low* vs *high CES-D score among women*
% *Depressive symptoms*									
CES-D, mean±s.e.m.	**8.3±0.2**	**24.5±0.4**	**14.2±0.3**	**7.9±0.2**	**25.8±0.3**	**16.2±0.3**	**<0.001**	**<0.001**	**<0.001**
Total WBC, K mm^−3^, mean±s.e.m.	6.37±0.09	6.42±0.12	**6.38±0.07**	**6.45±0.09**	**6.76±0.10**	**6.60±0.07**	**0.030**	0.73	**0.02**
10th – 90th percentile	82.6	76.5	80.3	81.0	78.6	79.9	0.36	***0.09***	0.44
<10th percentile: <4.1	10.3	14.11	11.7	10.4	10.6	10.5			
>90th percentile: >9.5	7.2	9.4	8.0	8.6	10.8	9.6			
									
% Neutrophils, mean±s.e.m.	57.4±0.5	56.2±0.6	**56.9±0.4**	**58.6±0.3**	**57.7±0.4**	**58.2±0.3**	**0.007**	0.15	0.13
10th–90th percentile	79.0	77.4	**78.4**	**85.7**	**79.6**	**82.7**	**0.002**	0.40	**0.019**
<10th percentile: <44.1	11.5	14.4	**12.6**	**5.9**	**9.9**	**7.8**			
>90th percentile: >70.8	9.5	8.2	**9.0**	**8.6**	**10.6**	**9.5**			
									
% Lymphocytes, mean±s.e.m.	32.0±0.4	32.9±0.6	32.4±0.3	32.2±0.3	32.9±0.4	32.5±0.3	0.66	0.23	0.19
10th–90th percentile	77.3	75.9	**76.8**	84.8	80.1	**83.0**	**0.002**	0.70	0.16
<10th percentile: <20.8	11.7	11.3	**11.5**	7.6	8.5	**8.0**			
>90th percentile: >44.7	11.0	12.9	**11.7**	7.6	10.6	**9.0**			
									
*Socio-demographic, lifestyle and health-related factors*									
Age (y), mean±s.e.m.	48.7±0.4	48.1±0.5	48.5±0.31	48.5±0.4	48.1±0.4	48.3±0.3	0.61	0.34	0.42
African American, *%*	**55.4**	**63.0**	58.2	57.3	54.9	56.2	0.37	**0.028**	0.43
Education, *%*							0.27	**<0.001**	**<0.001**
<HS	**7.1**	**9.1**	7.8	**4.8**	**7.4**	6.0			
HS	**54.0**	**68.0**	59.1	**54.3**	**63.1**	58.4			
>HS	**39.0**	**22.9**	33.1	**40.9**	**29.4**	35.5			
Missing	**0.0**	**0.0**	0.1	**0.0**	**0.2**	0.0			
PIR⩾125%, *%*	**66.4**	**51.4**	**60.9**	**61.9**	**46.4**	**54.7**	**0.005**	**<0.001**	**<0.001**
Employed, *%*							**<0.001**	**<0.001**	**<0.001**
Yes	**57.0**	**37.9**	**50.1**	**51.2**	**33.1**	**42.8**			
Missing	**17.5**	**21.3**	**18.9**	**18.0**	**17.6**	**17.8**			
									
Current smoking status, *%*							**<0.001**	**0.022**	**<0.001**
Currently smoking	**47.7**	**56.4**	**50.9**	**32.2**	**50.8**	**40.8**			
Missing	**2.9**	**3.8**	**3.2**	**5.3**	**6.1**	**5.6**			
									
Current use of illicit drugs, *%*							**<0.001**	0.39	**0.002**
Used any type	57.0	58.6	**57.6**	**30.0**	**40.2**	**34.7**			
Missing	5.9	7.8	**6.6**	**8.8**	**7.0**	**7.9**			
Body mass index, kg/m^2^; mean±s.e.m.	28.3±0.3	27.7±0.4	**28.0±0.2**	31.4±0.3	31.3±0.4	**31.3±0.3**	**<0.001**	0.17	0.90
									
*Dietary factors, daily intakes*									
Energy, kcal	2410±46	2295±62	**2369±38**	1719±29	1753±35	**1735±22**	**0.027**	0.13	0.46
Total carotenoids, mg per 1000 kcal	**3991±200**	**3161±207**	3689±149	**4494±194**	**3739±196**	4142±149	0.19	**0.007**	**0.007**
Vitamin A, RE per 1000 kcal	313±23	308±30	**311±18**	350±18	337±30	**344±17**	**0.013**	0.91	0.70
Vitamin C, mg per 1000 kcal	37.3±1.6	33.7±2.3	**36.0±1.3**	**42.1±1.6**	**38.6±2.0**	**40.5±1.3**	**<0.001**	0.18	0.16
Vitamin E, mg per 1000 kcal	**3.2±0.1**	**2.9±0.1**	3.1±0.1	**3.7±0.1**	**3.3±0.1**	3.5±0.1	0.47	**0.026**	**0.008**
Vitamin B-6, mg per 1000 kcal	***0.95±0.02***	***0.89±0.02***	0.93±0.02	**0.95±0.02**	**0.87±0.02**	0.91±0.01	0.58	*0.09*	**0.003**
Vitamin B-12, μg per 1000 kcal	3.15±0.25	3.17±0.29	**3.16±0.19**	3.00±0.19	3.04±0.29	**3.02±0.17**	**0.035**	0.96	0.91
Folate, μg per 1000 kcal	181.7±4.4	173.8±4.8	179.0±3.3	***193.3±4.1***	***182.1±4.1***	188.1±3.0	0.85	0.25	*0.05*
n-3 PUFA:n-6 PUFA ratio^c^	0.115±0.004	0.111±0.002	**0.113±0.003**	***0.117±0.002***	***0.111±0.002***	**0.114±0.002**	**0.027**	0.49	*0.07*
Healthy Eating Index-2010	**42.6±0.5**	**40.1±0.55**	**41.7±0.4**	**45.2±0.51**	**41.2±0.5**	**43.5±0.4**	**0.002**	**0.001**	**<0.001**

Abbreviations: AA, arachidonic acid; ALA, α-linolenic acid; CES-D, Center for Epidemiologic Studies-Depression scale; DHA, docosahexaenoic acid; DPA, docosapentaenoic acid; EPA, eicosapentaenoic acid; HANDLS, healthy aging in neighborhoods of diversity across the lifespan; HDL-C, high-density lipoprotein-cholesterol; HS, high school; LA, linoleic acid; n-3, omega-3; n-6, omega-6; PIR, poverty income ratio; PUFA, polyunsaturated fatty acid; TC, total cholesterol; WBC, white blood cell.

c n-3 PUFA included DHA+EPA+n-3DPA+ALA. n-6 PUFA included AA+LA.

Different from corresponding 'Men', *P<0.05. Different from corresponding 'CES-D score <16', ^#^*P*<0.05.

**Table 2:** Analysis of baseline total WBC count and WBC composition (% lymphocyte; % neutrophil) exposures and longitudinal change in CES-D score (sex-stratified), mixed-effects linear regression analysis, HANDLS study, 2004–2013

	*Men: Model 2*^a^		*Women: Model 3*^a^	
	*γ±SEE*	P*-value*	*γ±SEE*	P*-value*
	N *=*877	N *'=1488*	N *=1134*	N *'=2008*
*Total WBC count (TWBCC)*				
*Fixed effect*				
Intercept (γ_00_ for π_0i_)	**+19.50±2.29**	**<0.001**	**+20.18±2.12**	**<0.001**
Time (γ_10_ for π_1i_)	+0.50±0.52	0.34	−0.13±0.49	0.79
Age_base_	**−0.08±0.04**	**0.031**	**−0.07±0.04**	**0.034**
Age_base_ × time	***+0.02*±*0.01***	***0.08***	+0.013±0.008	0.13
				
*TWBCC (γ_011a_ for π_0i_)*				
<10th vs 10th−90th	**+2.51±1.05**	**0.016**	−0.08±1.09	0.94
>90th vs 10th−90th	−0.47±1.23	0.70	−1.13±1.16	0.33
				
*TWBCC × time (γ_111a_ for π_1i_)*				
<10th vs 10th−90th	−*0.44*±*0.23*	*0.06*	+0.11±0.24	0.65
>90th vs 10th−90th	+0.40±0.29	0.17	**+0.81±0.28**	**0.003**
				
*Random effects*				
Level 1 residuals (R_ij_)	**+6.07**±**0.27**	**<0.001**	**+7.60**±**0.18**	**<0.001**
* Level 2 residuals*				
Intercept (ξ_0i_)	**+7.32**±**0.27**	**<0.001**	**+8.05**±**0.27**	**<0.001**
Linear slope (ξ_1i_)	+0.45±0.26	NS	+0.00±0.06	NS

*Percent neutrophil (PN)*				
*Fixed effect*				
Intercept (γ_00_ for π_0i_)	**+20.3**±**2.29**	**<0.001**	**+19.77**±**2.11**	**<0.001**
Time (γ_10_ for π_1i_)	**+0.39**±**0.52**	**0.027**	−0.13±0.49	0.79
Age_base_	−**0.08**±**0.04**	**0.027**	−**0.08**±**0.04**	**0.035**
Age_base_ × time	***+0.02***±***0.01***	***0.09***	+0.012±0.009	0.15
				
*PN (γ_012a_ for π_0i_)*				
<10th vs 10th−90th	−0.10±1.03	0.93	**+2.70**±**1.26**	**0.031**^b^
>90th vs 10th−90th	−0.59±1.16	0.61	+1.60±1.14	0.16

*PN × time (γ_112a_ for π_1i_)*				
<10th vs 10th−90th	−0.10±0.24	0.70	+0.07±0.28	0.79
>90th vs 10th−90th	−0.11±0.26	0.69	+0.05±0.28	0.86
				
*Random effects*				
Level 1 residuals (R_ij_)	**+6.11**±**0.27**	**<0.001**	**+7.63**±**0.18**	**<0.001**
*Level 2 residuals*				
Intercept (ξ_0i_)	**+7.33**±**0.27**	**<0.001**	**+8.00**±**0.27**	**<0.001**
Linear slope (ξ_1i_)	+0.42±0.27	NS	+0.00±0.00	NS

*Percent lymphocytes (PL)*				
*Fixed effect*				
Intercept (γ_00_ for π_0i_)	**+20.18**±**2.29**	**<0.001**	**+19.72**±**2.12**	**<0.001**
Time (γ_10_ for π_1i_)	+0.38±0.52	0.46	−0.09±0.49	0.86
Age_base_	−**0.08**±**0.04**	**0.025**	−**0.079**±**0.036**	**0.030**
Age_base_ × time	***+0.016***±***0.009***	***0.08***	+0.012±0.009	0.15

*PL (γ_013a_ for π_0i_)*				
<10th vs 10th−90th	+0.17±1.04	0.87	+1.95±1231	0.11
>90th vs 10th−90th	+0.74±1.06	0.69	+1.90±1.18	0.11

* PL × time (γ_113a_ for π_1i_)*				
<10th vs 10th−90th	−0.16±0.25	0.53	−0.26±0.29	0.38
>90th vs 10th−90th	−0.11±0.25	0.65	+0.001±0.27	0.99

*Random effects*				
Level 1 residuals (R_ij_)	**+6.11**±**0.27**	**<0.001**	**+7.63**±**0.18**	**<0.001**
* Level 2 residuals*				
Intercept (ξ_0i_)	**+7.33**±**0.27**	**<0.001**	**+8.01**±**0.28**	**<0.001**
Linear slope (ξ_1i_)	+0.43±0.27	NS	+0.001±0.001	NS

Abbreviations: AA, arachidonic acid; ALA, α-linolenic acid; CES-D, Center for Epidemiologic Studies-Depression scale; DHA, docosahexaenoic acid; DPA, docosapentaenoic acid; EPA, eicosapentaenoic acid; HANDLS, healthy aging in neighborhoods of diversity across the lifespan; HDL-C, high-density lipoprotein-cholesterol; HS, high school; LA, linoleic acid; LDL-C, low-density lipoprotein-cholesterol; n-3, omega-3; n-6, omega-6; NS, not significant; PUFA, polyunsaturated fatty acids; TC, total cholesterol; WBC, white blood cell.

a Models were further adjusted for other covariates (main effects and interaction with time). See the 'Materials and methods' section for more details on covariate coding and model specifications. Time at baseline visit was set to zero. Baseline age was centered at 50 years (y), total energy intake at 2000 kcal per day, total carotenoid intake at 3 mg per 1000 kcal per day, vitamin C intake at 30 mg per 1000 kcal per day, vitamin A intake at 300 RE per 1000 kcal per day, vitamin E at 3 mg per 1000 kcal per day, vitamin B-6 at 0.8 mg per 1000 kcal per day, vitamin B-12 at 3 μg per 1000 kcal per day, folate at 170 μg per 1000 kcal per day, n-3 PUFA:n-6 PUFA at 0.11. Healthy Eating Index-2010 was centered at 42.

b In a separate model with interaction of WBC exposures by sex by time, including all other terms in the current model, *P*<0.10 for null hypothesis that this interaction term is =0.

*n* represents the number of participants in the analysis; *n*' represents the total number of visits included in the analysis. Findings that were significant at a type I error of 0.05 are in bold. Subscript 'a' refers to the exposure level whereby 1: <10th vs 10th−90th and 2: >90th vs 10th−90th. Italic entries are when *P*<0.10 but >0.05, so marginally significant results prior to correction for multiple testing.

**Table 3:** Analysis of baseline total WBC count, % neutrophil (PN), % lymphocytes (PL) and longitudinal change in CES-D component scores among women, mixed-effects linear regression analysis, HANDLS study, 2004–2013

	*X* = *Total WBC count*		*X* = *PN*		*X* = *PL*	
	*γ±SEE*	P*-value*	*γ±SEE*	P*-value*	*γ±SEE*	P*-value*
	N*=1134*	N *'=2011*	N *=1134*	N *'=2011*	N *=1134*	N *'=2011*
*Y* = *CES-D component 1: somatic complaints*						
* Fixed effect*						
Intercept (γ_00_ for π_0i_)	**+8.63±0.82**	**<0.001**	**+8.42±0.81**	**<0.001**	**+8.37±0.81**	**<0.001**
Time (γ_10_ for π_1i_)	+0.08±0.20	0.68	+0.10±0.20	0.63	+0.11±0.01	0.18
Age_base_	−0.018±0.014	0.21	−0.02±0.01	0.20	−0.019±0.014	0.18
Age_base_ × time	+0.003±0.004	0.36	+0.003±0.004	0.40	+0.003±0.004	0.36
* WBC exposure (γ*_*01ea*_ *for π*_*0i*_)						
<10th vs 10th–90th	−0.18±0.42	0.66	**+1.61±0.48**	**0.001**^a^	***+0.88*±*0.47***	***0.06***
>90th vs 10th–90th	−0.26±0.45	0.56	+0.57±0.44	0.19	**+1.16±0.45**	**0.011**^a^
						
* WBC exposure × time (γ*_*11ea*_ *for π*_*1i*_)						
<10th vs 10th–90th	+0.03±0.10	0.73	**−*0.19*±*0.12***	***0.097***	−0.17±0.12	0.15
>90th vs 10th–90th	**+0.25±0.11**	**0.029**	−0.07±0.12	0.53	−0.16±0.11	0.16

* Random effects*						
Level 1 residuals (R_ij_)	**+3.19±0.07**	**<0.001**	**+3.19±0.08**	**<0.001**	**+3.19±0.08**	**<0.001**
*Level 2 residuals*						
Intercept (ξ_0i_)	**+2.85±0.11**	**<0.001**	**+2.83±0.11**	**<0.001**	**+2.83±0.11**	**<0.001**
Linear slope (ξ_1i_)	+0.00±0.01	NS	+0.00±0.00	NS	0.00±0.00	NS
						
*Y* = *CES-D component 2: depressed affect*						
*Fixed effect*						
Intercept (γ_00_ for π_0i_)	**+6.67±0.93**	**<0.001**	**+6.52±0.93**	**<0.001**	**+6.57±0.93**	**<0.001**
Time (γ_10_ for π_1i_)	−0.08±0.22	0.71	−0.11±0.22	0.63	−0.100±0.225	0.66
Age_base_	**−*0.028*±*0.016***	***0.09***	**−*0.03*±*0.02***	***0.09***	**−*0.027*±*0.016***	***0.09***
Age_base_ × time	+0.004±0.004	0.23	+0.005±0.004	0.24	+0.004±0.004	0.28
* WBC exposure (γ_01ea_ for π_0i_)*						
<10th vs 10th–90th	−0.11±0.48	0.83	+0.42±0.56	0.45	+0.39±0.54	0.47
>90th vs 10th–90th	−0.36±0.51	0.49	+0.59±0.50	0.25	−0.01±0.52	0.98

* WBC exposure × time (γ*_*11ea*_ *for π*_*1i*_)						
<10th vs 10th–90th	+0.02±0.11	0.85	+0.16±0.13	0.20	−0.034±0.135	0.80
>90th vs 10th–90th	**+0.33±0.13**	**0.009**	+0.09±0.13	0.49	+0.155±0.123	0.21
* Random effects*						
Level 1 residuals (R_ij_)	**+3.42±0.12**	**<0.001**	**+3.43±0.12**	**<0.001**	**+3.42±0.12**	**<0.001**
*Level 2 residuals*						
Intercept (ξ_0i_)	**+3.51±0.13**	**<0.001**	**+3.49±0.13**	**<0.001**	**+3.50±0.13**	**<0.001**
Linear slope (ξ_1i_)	**+0.21±0.13**	**<0.05**	+0.22±0.13	NS	+0.22±0.12	NS
						
*Y* = *CES-D component 3: positive affect*						
*Fixed effect*						
Intercept (γ_00_ for π_0i_)	**+8.53±0.50**	**<0.001**	**+8.56±0.50**	**<0.001**	**+8.61±0.50**	**<0.001**
Time (γ_10_ for π_1i_)	+0.04±0.12	0.69	+0.03±0.12	0.81	+0.02±0.12	0.87
Age_base_	**+0.023±0.009**	**0.007**	**+0.023±0.010**	**0.008**	**+0.02±0.01**	**0.006**
Age_base_ × time	−0.001±0.002	0.53	−0.0011±0.0021	0.61	−0.001±0.002	0.56
* WBC exposure (γ_01ea_ for π_0i_)*						
<10th vs 10th–90th	−0.19±0.26	0.47	**−0.60±0.30**	**0.047**	**−*0.55*±*0.29***	***0.06***^a^
>90th vs 10th–90th	**+0.55±0.28**	**0.047**	−0.34±0.27	0.21^a^	**−0.69±0.28**	**0.017**

* WBC exposure × time (γ*_*11ea*_ *for π*_*1i*_)						
<10th vs 10th–90th	−0.04±0.06	0.55	−0.068±0.069	0.33	+0.12±0.07	0.10
>90th vs 10th–90th	**−0.17±0.07**	**0.012**	+0.107±0.070	0.12	−0.02±0.07	0.76

* Random effects*						
Level 1 residuals (R_ij_)	**+1.88±0.04**	**<0.001**	**+1.88±0.05**	**<0.001**	**+1.88±0.05**	**<0.001**
* Level 2 residuals*						
Intercept (ξ_0i_)	**+1.85±0.07**	**<0.001**	**+1.85±0.07**	**<0.001**	**+1.84±0.07**	**<0.001**
Linear slope (ξ_1i_)	**+0.00±0.00**	NS	0.00±0.00	NS	+0.000±0.014	NS

*Y* = *CES-D component 4: interpersonal problems*						
*Fixed effect*						
Intercept (γ_00_ for π_0i_)	**+1.28±0.25**	**<0.001**	**+1.27±0.25**	**<0.001**	**+1.27±0.25**	**<0.001**
Time (γ_10_ for π_1i_)	−0.06±0.07	0.38	+0.06±0.07	0.33	−0.055±0.066	0.41
Age_base_	**−0.008±0.004**	**0.048**	**−*0.008*±*0.004***	***0.05***	**−0.008±0.004**	**0.047**
Age_base_ × time	**+0.003±0.001**	**0.007**	**+0.003±0.001**	**0.007**	**+0.003±0.001**	**0.008**
						
* WBC exposure (γ_01ea_ for π_0i_)*						
<10th vs 10th–90th	+0.037±0.128	0.77	+0.07±0.15	0.64	+0.13±0.14	0.38
>90th vs 10th–90th	−0.052±0.136	0.71	+0.08±0.14	0.54	+0.08±0.14	0.58
* WBC exposure × time (γ_11ea_ for π_1i_)*						
<10th vs 10th–90th	+0.010±0.032	0.75	+0.036±0.038	0.35	−0.01±0.04	0.72
>90th vs 10th–90th	***+0.071*±*0.080***	***0.062***	+0.038±0.038	0.33	−0.02±0.04	0.61

*Random effects*						
Level 1 residuals (R_ij_)	**+0.96±0.03**	**<0.001**	**+0.96±0.04**	**<0.001**	**+0.96±0.03**	**<0.001**
*Level 2 residuals*						
Intercept (ξ_0i_)	**+0.87±0.04**	**<0.001**	**+0.87±0.04**	**<0.001**	**+0.87±0.04**	**<0.001**
Linear slope (ξ_1i_)	**+0.11±0.02**	**<0.001**	**+0.11±0.02**	**<0.001**	**+0.11±0.02**	**<0.001**

Abbreviations: AA, arachidonic acid; ALA, α-linolenic acid; CES-D, Center for Epidemiologic Studies-Depression scale; DHA, docosahexaenoic acid; DPA, docosapentaenoic acid; EPA, eicosapentaenoic acid; HANDLS, healthy aging in neighborhoods of diversity across the lifespan; HS, high school; HUFA, highly unsaturated fatty acids; LA, linoleic acid; n-3, mega-3; n-6, omega-6; NS, not significant; PUFA, polyunsaturated fatty acids; WBC, white blood cell.

a In a separate model with interaction of WBC exposures by sex by time, including all other terms in the current model, *P*<0.10 for null hypothesis that this interaction term is =0.

Models were further adjusted for other covariates (main effects and interaction with time). See the 'Materials and methods' section for more details on covariate coding and model specifications. Time at baseline visit was set to zero. Baseline age was centered at 50 years (y), total energy intake at 2000 kcal per day, total carotenoid intake at 3 mg per 1000 kcal per day, vitamin C intake at 30 mg per 1000 kcal per day, vitamin A intake at 300 RE per 1000 kcal per day, vitamin E at 3 mg per 1000 kcal per day, vitamin B-6 at 0.8 mg per 1000 kcal per day, vitamin B-12 at 3 μg per 1000 kcal per day, folate at 170 μg per 1000 kcal per day, n-3 PUFA:n-6 PUFA at 0.11. Healthy Eating Index-2010 was centered at 42. *n* represents the number of participants in the analysis; *n*' represents the total number of visits included in the analysis. Findings that were significant at a type I error of 0.05 are in bold. Subscript 'e' refers to exposure whereby 1: TWBCC, 2: PN, 3: PL; 'a' refers to the exposure level whereby 1: <10th vs 10th−90th and 2: >90th vs 10th−90th. Italic entries are when *P*<0.10 but >0.05, so marginally significant results prior to correction for multiple testing.

**Table 4:** Analysis of baseline total WBC count, % neutrophil (PN),% lymphocyte (PL) and longitudinal change in CES-D component scores among men, mixed-effects linear regression analysis, HANDLS study, 2004–2013

	*X* = *Total WBC count*		*X* = *PN*		*X* = *PL*	
	*γ±SEE*	P*-value*	*γ±SEE*	P*-value*	γ*±*SEE	P-value
	N *=*877	N *'=1490*	N *=*877	N *'=1490*	N *=*877	N *'=1490*
*Y* = *CES-D component 1: somatic complaints*						
* Fixed effect*						
Intercept (γ_00_ for π_0i_)	**+8.18±0.90**	**<0.001**	**+8.30±0.90**	**<0.001**	**+8.23±0.90**	**<0.001**
Time (γ_10_ for π_1i_)	+0.16±0.22	0.48	+0.14±0.22	0.51	+0.14±0.21	0.51
Age_base_	**−*0.025*±*0.015***	***0.09***	**−*0.026*±*0.015***	***0.08***	**−*0.03*±*0.01***	***0.07***
Age_base_ × time	+0.003±0.004	0.35	+0.003±0.004	0.40	−0.027±0.015	0.51
* WBC exposure (γ*_*01ea*_ *for π*_*0i*_)						
<10th vs 10th −90th	+0.34±0.41	0.41	−0.25±0.40	0.53	+0.27±0.41	0.51
>90th vs 10th −90th	+0.32±0.48	0.51	+0.20±0.46	0.66	+0.16±0.42	0.70
						
* WBC exposure × time (γ*_*11ea*_ *for π*_*1i*_)						
<10th vs 10th −90th	−0.05±0.10	0.59	−0.01±0.10	0.93	−0.07±0.11	0.49
>90th vs 10th −90th	+0.16±0.12	0.18	−0.08±0.11	0.47	−0.03±0.10	0.77
						
* Random effects*						
Level 1 residuals (R_ij_)	**+2.59±0.11**	**<0.001**	**+2.60±0.11**	**<0.001**	**+2.60±0.11**	**<0.001**
* Level 2 residuals*						
Intercept (ξ_0i_)	**+2.71±0.11**	**<0.001**	**+2.71±0.11**	**<0.001**	**+2.71±0.11**	**<0.001**
Linear slope (ξ_1i_)	+0.17±0.11	NS	+0.18±0.11	NS	+0.18±0.11	NS
						
*Y* = *CES-D component 2: depressed affect*						
*Fixed effect*						
Intercept (γ_00_ for π_0i_)	**+6.47±0.99**	**<0.001**	**+6.87±0.11**	**<0.001**	**+6.78±0.98**	**<0.001**
Time (γ_10_ for π_1i_)	+0.04±0.20	0.86	−0.02±0.23	0.92	−0.03±0.24	0.90
Age_base_	−0.016±0.016	0.32	−0.017±0.016	0.29	−0.02±0.02	0.27
Age_base_ × time	+0.004±0.004	0.29	+0.004±0.004	0.31	+0.004±0.004	0.32
*WBC exposure (γ*_*01ea*_ *for π*_*0i*_)						
<10th vs 10th−90th	**+1.17±0.45**	**0.009**	−0.11±0.45	0.81	+0.20±0.45	0.65
>90th vs 10th−90th	−0.37±0.53	0.49	−0.27±0.50	0.59	+0.19±0.46	0.67
						
* WBC exposure × time (γ*_*11ea*_ *for π*_*1i*_)						
<10th vs 10th−90th	**−0.22±0.10**	**0.035**	+0.04±0.11	0.73	−0.00±0.11	0.97
>90th vs 10th−90th	*+0.22*±*0.13*	*0.09*	−0.01±0.13	0.93	−0.02±0.11	0.84
						
*Random effects*						
Level 1 residuals (R_ij_)	**+2.80±0.12**	**<0.001**	**+2.82±0.12**	**<0.001**	**+2.82±0.10**	**<0.001**
*Level 2 residuals*						
Intercept (ξ_0i_)	**+3.02±0.12**	**<0.001**	**+3.02±0.13**	**<0.001**	**+3.03±0.12**	**<0.001**
Linear slope (ξ_1i_)	**+0.21±0.11**	**<0.001**	**+0.20±0.11**	**<0.05**	+0.20±0.12	NS
						
*Y* = *CES-D component 3: positive affect*						
*Fixed effect*						
Intercept (γ_00_ for π_0i_)	**+8.82±0.56**	**<0.001**	**+8.57±0.56**	**<0.001**	**+8.54±0.56**	**<0.001**
Time (γ_10_ for π_1i_)	**−*0.28*±*0.15***	***0.06***	−0.23±0.15	0.11	−0.24±0.14	0.10
Age_base_	**+0.024±0.009**	**0.008**	**+0.026±0.009**	**0.006**	**+0.02±0.01**	**0.009**
Age_base_ × time	**−0.006±0.003**	**0.023**	**−0.006±0.003**	**0.020**	**−0.006±0.002**	**0.019**
* WBC exposure (γ*_*01ea*_ *for π*_*0i*_)						
<10th vs 10th−90th	**−0.78±0.26**	**0.003**	−0.32±0.25	0.21	+0.33±0.26	0.20
>90th vs 10th−90th	+0.26±0.30	0.38	***+0.52*****±*0.28***	*0.07*	−0.34±0.26	0.19
						
* WBC exposure × time (γ*_*11ea*_ *for π*_*1i*_)						
<10th vs 10th−90th	**+0.15±0.06**	**0.021**	+0.008±0.068	0.91	+0.03±0.07	0.71
>90th vs 10th−90th	−0.03±0.08	0.69	−0.024±0.076	0.75	−0.01±0.07	0.93
						
*Random effects*						
Level 1 residuals (R_ij_)	**+1.68±0.07**	**<0.001**	**+1.69±0.07**	**<0.001**	**+1.70±0.07**	**<0.001**
*Level 2 residuals*						
Intercept (ξ_0i_)	**+1.64±0.08**	**<0.001**	**+1.63±0.07**	**<0.001**	**+1.63±0.08**	**<0.001**
Linear slope (ξ_1i_)	**+0.16±0.05**	**<0.001**	**+0.15±0.05**	**<0.001**	**+0.15±0.05**	**<0.001**
						
*Y* = *CES-D component 4: interpersonal problems*						
*Fixed effect*						
Intercept (γ_00_ for π_0i_)	**+1.62±0.32**	**<0.001**	**+1.70±0.32**	**<0.001**	**+1.69±0.32**	**<0.001**
Time (γ_10_ for π_1i_)	+0.05±0.09	0.60	+0.05±0.09	0.59	+0.04±0.08	0.60
Age_base_	**−0.015±0.005**	**0.006**	**−0.015±0.005**	**0.006**	**−0.015±0.005**	**0.006**
Age_base_ × time	+0.0022±0.0015	0.13	+0.002±0.002	0.10	+0.002±0.001	0.10
*WBC exposure (γ*_*01ea*_ *for π*_*0i*_)						
<10th vs 10th−90th	+0.23±0.15	0.12	−0.04±0.15	0.76	+0.04±0.15	0.79
>90th vs 10th−90th	−0.17±0.17	0.33	+0.02±0.16	0.93	+0.04±0.15	0.80
						
* WBC exposure × time (γ*_*11ea*_ *for π*_*1i*_)						
<10th vs 10th−90th	−0.03±0.04	0.49	−0.033±0.040	0.41	−0.04±0.04	0.31
>90th vs 10th−90th	−0.00±0.05	0.93	−0.024±0.045	0.58	−0.06±0.04	0.17
						
*Random effects*						
Level 1 residuals (R_ij_)	**+1.04±0.04**	**<0.001**	**+1.04±0.04**	**<0.001**	**+1.04±0.04**	**<0.001**
* Level 2 residuals*						
Intercept (ξ_0i_)	**+0.86±0.05**	**<0.001**	**+0.86±0.05**	**<0.001**	**+0.86±0.05**	**<0.001**
Linear slope (ξ_1i_)	+0.05±0.05	NS	+0.05±0.06	NS	+0.05±0.06	NS

Abbreviations: AA, arachidonic acid; ALA, α-linolenic acid; CES-D, Center for Epidemiologic Studies-Depression scale; DHA, docosahexaenoic acid; DPA, docosapentaenoic acid; EPA, eicosapentaenoic acid; HANDLS, healthy aging in neighborhoods of diversity across the lifespan; HS, high school; HUFA, highly unsaturated fatty acids; LA, linoleic acid; n-3, omega-3; n-6, omega-6; NS, not significant; PIR, poverty income ratio; PUFA, polyunsaturated fatty acids; WBC, white blood cell.

Models were further adjusted for other covariates (main effects and interaction with time). See the 'Materials and methods' section for more details on covariate coding and model specifications. Time at baseline visit was set to zero. Baseline age was centered at 50 years (y), total energy intake at 2000 kcal per day, total carotenoid intake at 3 mg per 1000 kcal per day, vitamin C intake at 30 mg per 1000 kcal per day, vitamin A intake at 300 RE per 1000 kcal per day, vitamin E at 3 mg per 1000 kcal per day, vitamin B-6 at 0.8 mg per 1000 kcal per day, vitamin B-12 at 3 μg per 1000 kcal per day, folate at 170 μg per 1000 kcal per day, n-3 PUFA:n-6 PUFA at 0.11. Healthy Eating Index-2010 was centered at 42. *n* represents the number of participants in the analysis; *n*' represents the total number of visits included in the analysis. Findings that were significant at a type I error of 0.05 are in bold. Subscript 'e' refers to exposure whereby 1: TWBCC, 2:PN, 3:PL; 'a' refers to the exposure level whereby 1: <10th vs 10th−90th and 2: >90th vs 10th−90th. Italic entries are when *P*<0.10 but >0.05, so marginally significant results prior to correction for multiple testing.
