# Supplementary material for: White blood cell inflammatory markers are associated with depressive symptoms in a longitudinal study of urban adults
Source: Transl Psychiatry. 2016 Sep 20;6(9):e895–. doi: 10.1038/tp.2016.180 (PMC5048214; doi:10.1038/tp.2016.180)
Supplement: Supplementary Information [file tp2016180x1.doc]

**OSM 1: Mixed-effects regression models**

The mixed-effects regression models can be summarized as follows:

**Multi-level models** vs. **Composite models**

| **Eq.**  **1.1-1.4** | $Y_{ij}=\pi_{0i}+\pi_{1i}Time_{ij}+\varepsilon_{ij}$ |  |  |
| --- | --- | --- | --- |

Where Y_ij_ is the outcome (CES-D total or domain-specific score) for each individual “i” and visit “j”; $\pi_{0i}$is the level-1 intercept for individual i; $\pi_{1i}$is the level-1 slope for individual i; $\gamma_{00}$is the level-2 intercept of the random intercept $\pi_{0i}$; $\gamma_{10}$is the level-2 intercept of the slope $\pi_{1i}$; $Z_{ik}$is a vector of fixed covariates for each individual *i* that are used to predict level-1 intercepts and slopes and included baseline age (Age_base_) among other covariates. X_ija_, represents the main predictor variables (TWBCC, PL and PN); $\zeta_{0i}$and $\zeta_{1i}$are level-2 disturbances; $\varepsilon_{ij}$is the within-person level-1 disturbance. Of primary interest are the main effects of each exposure X_a_ (γ_0a_) and their interaction with *TIME* (γ_1a_), as described in a previous methodolgical paper.[^58^](#_ENREF_58)

**TABLE S1.** Analysis of baseline *CES-D score* (X) and longitudinal change in total WBC count, % lymphocytes and % neutrophils: sex-stratified mixed-effects linear regression analysis, HANDLS study, 2004-2013

|  | **Both genders: Model 1**^1^ | | **Women: Model 2**^1^ | | **Men: Model 3** ^1^ | | |  | |
| --- | --- | --- | --- | --- | --- | --- | --- | --- | --- |
|  | γ±SEE | p-value | γ±SEE | p-value | γ±SEE | p-value | |  | |
| Y=TWBCC | N=2,081 | N’=3,560 | N=1,173 | N’=2,041 | N=908 | N’=1,519 | |  | |
| ***Fixed effect*** |  |  |  |  |  |  | |  | |
| Intercept (γ_00_ for π_0i_) | **+4.88±0.31** | **<0.001** | **+4.65±0.41** | **<0.001** | **+4.89±0.47** | **<0.001** | |  | |
| Time (γ_10_ for π_1i_) | +0.04±0.06 | 0.49 | +0.07±0.08 | 0.39 | -0.01±0.10 | 0.91 | |  | |
| Age_base_ | -0.003±0.004 | 0.52 | -0.004±0.007 | 0.56 | -0.003±0.007 | 0.64 | |  | |
| Age_base_ ×Time | +0.000±0.001 | 0.49 | -0.002±0.001 | 0.17 | +0.002±0.002 | 0.22 | |  | |
| **CES-D** (γ_01_ for π_0i_) | -0.001±0.004 | 0.76 | +0.002±0.005 | 0.66 | -0.007±0.007 | 0.32 | |  | |
| **CES-D** ×Time (γ_11_ for π_1i_) | +0.0001±0.0009 | 0.94 | -0.0004±0.0011 | 0.71 | -0.000±0.001 | 0.82 | |  | |
| ***Random effects*** |  |  |  |  |  |  | |  | |
| Level 1 residuals (R_ij_) | **+1.15±0.04** | **<0.001** | **+1.10±0.05** | **<0.001** | **+1.20±0.05** | **<0.001** | |  | |
| Level 2 residuals |  |  |  |  |  |  | |  | |
| Intercept (ξ_0i_) | **+1.62±0.04** | **<0.001** | **+1.70±0.05** | **<0.001** | **+1.47±0.05** | **<0.001** | |  | |
| Linear slope (ξ_1i_) | **+0.10±0.03** | **<0.001** | **+0.13±0.03** | **<0.001** | +0.016±0.24 | ns | |  | |
|  |  |  |  |  |  |  | |  | |
| **Y=PN** | N=2,081 | N’=3,560 | N=1,173 | N’=2,041 | N=908 | N’=1,514 | |  | |
| ***Fixed effect*** |  |  |  |  |  |  | |  | |
| Intercept (γ_00_ for π_0i_) | **+61.2±1.56** | **<0.001** | **+61.9±1.9** | **<0.001** | **60.0±2.6** | **<0.001** | |  | |
| Time (γ_10_ for π_1i_) | ***+0.70±0.40*** | ***0.07*** | +0.39±0.49 | 0.87 | +1.06±0.64 | 0.10 | |  | |
| Age_base_ | ***-0.04±0.02*** | ***0.08*** | **-0.08±0.03** | **0.007** | -0.010±0.041 | 0.81 | |  | |
| Age_base_ ×Time | +0.004±0.006 | 0.51 | -0.001±0.008 | 0.87 | +0.010±0.011 | 0.32 | |  | |
| **CES-D** (γ_01_ for π_0i_) | -0.03±0.02 | 0.24 | -0.03±0.02 | 0.25 | -0.03±0.04 | 0.49 | |  | |
| **CES-D** ×Time (γ_11_ for π_1i_) | -0.005±0.005 | 0.38 | -0.004±0.006 | 0.51 | -0.007±0.009 | 0.47 | |  | |
| ***Random effects*** |  |  |  |  |  |  | |  | |
| Level 1 residuals (R_ij_) | **+6.76±0.20** | **<0.001** | **+6.26±0.26** | **<0.001** | **+7.41±0.33** | **<0.001** | |  | |
| Level 2 residuals |  |  |  |  |  |  | |  | |
| Intercept (ξ_0i_) | **+7.38±0.20** | **<0.001** | **+7.14±0.26** | **<0.001** | **+7.40±0.33** | **<0.001** | |  | |
| Linear slope (ξ_1i_) | **+0.81±0.12** | **<0.001** | **+0.96±0.12** | **<0.001** | +0.38±0.43 | ns | |  | |
|  |  |  |  |  |  |  | |  | |
| **Y=PL** | N=2,081 | N’=3,560 | N=1,173 | N’=2,041 | N=908 | N’=1,514 | |  | |
| ***Fixed effect*** |  |  |  |  |  |  | |  | |
| Intercept (γ_00_ for π_0i_) | **+29.07±1.41** | **<0.001** | **+28.87±1.72** | **<0.001** | **+27.83±2.35** | **<0.001** | |  | |
| Time (γ_10_ for π_1i_) | ***-0.58±0.33*** | ***0.08*** | -0.48±0.42 | 0.26 | -0.71±0.53 | 0.18 | |  | |
| Age_base_ | +0.017±0.022 | 0.46 | +0.04±0.03 | 0.13 | +0.016±0.037 | 0.66 | |  | |
| Age_base_ ×Time | -0.005±0.005 | 0.40 | +0.002±0.007 | 0.74 | -0.013±0.009 | 0.14 | |  | |
| **CES-D** (γ_01_ for π_0i_) | +0.021±0.019 | 0.26 | +0.023±0.023 | 0.31 | +0.027±0.033 | 0.41 | |  | |
| **CES-D** ×Time (γ_11_ for π_1i_) | +0.004±0.005 | 0.37 | +0.003±0.006 | 0.55 | +0.006±0.007 | 0.41 | |  | |
| ***Random effects*** |  |  |  |  |  |  | |  | |
| Level 1 residuals (R_ij_) | **+5.88±0.18** | **<0.001** | **+5.58±0.22** | **<0.001** | **+6.26±0.18** | **<0.001** | |  | |
| Level 2 residuals |  |  |  |  |  |  | |  | |
| Intercept (ξ_0i_) | **+6.83±0.17** | **<0.001** | **+6.53±0.22** | **<0.001** | **+6.97±0.27** | **<0.001** | |  | |
| Linear slope (ξ_1i_) | **+0.59±0.12** | **<0.001** | **+0.73±0.12** | **<0.001** | +0.000±0.001 | ns | |  | |
|  |  |  |  | | | |  | |  |

*Abbreviations*: AA=Arachidonic acid; ALA=α-linolenic acid; CES-D=Center for Epidemiologic Studies-Depression scale; DHA=Docosahexaenoic acid; DPA=Docosapentaenoic acid EPA=Eicosapentaenoic acid; HANDLS=Healthy Aging in Neighborhoods of Diversity Across the Lifespan; HS=High School; HUFA=highly unsaturated fatty acids; LA=Linoleic acid; *n3*=omega-3; *n6*=omega-6; PIR=Poverty Income Ratio; PUFA=polyunsaturated fatty acids; SEM=standard error of the mean.

^1^ Models were further adjusted for other covariates (main effects and interaction with time). See methods section for more details on covariate coding and model specifications. Time at baseline visit was set to zero. Baseline age was centered at 50y, total energy intake at 2000kcal/d, total carotenoid intake at 3mg/1,000kcal/d, vitamin C intake at 30mg/1,000kcal/d, vitamin A intake at 300 RE/1,000 kcal/d, vitamin E at 3 mg/1,000 kcal/d, vitamin B-6 at 0.8 mg/1,000 kcal/d, vitamin B-12 at 3 μg/1,000 kcal/d, folate at 170 μg/1,000 kcal/d, n-3 PUFA:n-6 PUFA at 0.11. Healthy Eating Index-2010 was centered at 42. ^2^ n=number of participants in the analysis; n’=total number of visits included in the analysis. Findings that were significant at a type I error of 0.05 are bolded. ^3^ In a separate model with interaction of CES-D by TIME by sex, including all other terms in the current model, p<0.10 for null hypothesis that this interaction term is=0.

**Table S2**. Key findings from Tables 2-4

|  | Men | Women | Interaction by sex |
| --- | --- | --- | --- |
| **Exposure 1: TWBCC** |  |  |  |
| ***CES-D change total*** |  |  |  |
| *TWBCC <10^th^ perc* | **+2.51, P=0.016** | ns | ns |
| *TWBCC <10^th^ perc×Time* | ***-0.44, p=0.060*** | ns | ns |
| *TWBCC >90^th^ perc* | ns | ns | ns |
| *TWBCC >90^th^ perc×Time* | ns | **+0.81, p=0.003** | ns |
|  |  |  |  |
| ***CES-D somatic complaints*** |  |  |  |
| *TWBCC <10^th^ perc* | ns | ns | ns |
| *TWBCC <10^th^ perc×Time* | ns | ns | ns |
| *TWBCC >90^th^ perc* | ns | ns | ns |
| *TWBCC >90^th^ perc×Time* | ns | **+0.25, p=0.029** | ns |
|  |  |  |  |
| ***CES-D depressed affect*** |  |  |  |
| *TWBCC <10^th^ perc* | **+1.17, p=0.009** | ns | ns |
| *TWBCC <10^th^ perc×Time* | ***-0.22, p=0.035*** | ns | ns |
| *TWBCC >90^th^ perc* | ns | ns | ns |
| *TWBCC >90^th^ perc×Time* | ***+0.22, p=0.09*** | **+0.33, p=0.009** | ns |
|  |  |  |  |
| ***CES-D positive affect*** |  |  |  |
| *TWBCC <10^th^ perc* | **-0.78, p=0.003** | ns | ns |
| *TWBCC <10^th^ perc×Time* | **+0.15, p=0.021** | ns | ns |
| *TWBCC >90^th^ perc* | ns | ***+0.55, p=0.047*** | ns |
| *TWBCC >90^th^ perc×Time* | ns | **-0.17, p=0.012** | ns |
|  |  |  |  |
| **CES-D Interpersonal problems** |  |  |  |
| *TWBCC <10^th^ perc* | ns | ns | ns |
| *TWBCC <10^th^ perc×Time* | ns | ns | ns |
| *TWBCC >90^th^ perc* | ns | ns | ns |
| *TWBCC >90^th^ perc×Time* | ns | ***+0.071, p=0.062*** | ns |
|  |  |  |  |
|  |  |  |  |
| **Exposure 2: PN** |  |  |  |
| ***CES-D change total*** |  |  |  |
| *PN <10^th^ perc* | ns | ***+2.71, p=0.031*** | P<0.10 |
| *PN <10^th^ perc×Time* | ns | ns | ns |
| *PN >90^th^ perc* | ns | ns | ns |
| *PN >90^th^ perc×Time* | ns | ns | ns |
|  |  |  |  |
| ***CES-D somatic complaints*** |  |  |  |
| *PN <10^th^ perc* | ns | **+1.61, p=0.001** | P<0.10 |
| *PN <10^th^ perc×Time* | ns | ***-0.19, P=0.097*** | ns |
| *PN >90^th^ perc* | ns | ns | ns |
| *PN >90^th^ perc×Time* | ns | ns | ns |
|  |  |  |  |
| ***CES-D depressed affect*** |  |  |  |
| *PN <10^th^ perc* | ns | ns | ns |
| *PN <10^th^ perc×Time* | ns | ns | ns |
| *PN >90^th^ perc* | ns | ns | ns |
| *PN >90^th^ perc×Time* | ns | ns | ns |
|  |  |  |  |
| ***CES-D positive affect*** |  |  |  |
| *PN <10^th^ perc* | ns | ***-0.60, p=0.047*** | ns |
| *PN <10^th^ perc×Time* | ns | ns | ns |
| *PN >90^th^ perc* | ns | ns | P<0.10 |
| *PN >90^th^ perc×Time* | ns | ns | ns |
|  |  |  |  |
| ***CES-D interpersonal problems*** |  |  |  |
| *PN <10^th^ perc* | ns | ns | ns |
| *PN <10^th^ perc×Time* | ns | ns | ns |
| *PN >90^th^ perc* | ns | ns | ns |
| *PN >90^th^ perc×Time* | ns | ns | ns |
|  |  |  |  |
| **Exposure 3: PL** |  |  |  |
| ***CES-D change total*** |  |  |  |
| *PL <10^th^ perc* | ns | ns | ns |
| *PL <10^th^ perc×Time* | ns | ns | ns |
| *PL >90^th^ perc* | ns | ns | ns |
| *PL >90^th^ perc×Time* | ns | ns | ns |
|  |  |  |  |
| ***CES-D somatic complaints*** |  |  |  |
| *PL <10^th^ perc* | ns | ***+0.88, p=0.06*** | ns |
| *PL <10^th^ perc×Time* | ns | ns | ns |
| *PL >90^th^ perc* | ns | **+1.16, p=0.011** | P<0.10 |
| *PL >90^th^ perc×Time* | ns | ns | ns |
|  |  |  |  |
| ***CES-D depressed affect*** |  |  |  |
| *PL <10^th^ perc* | ns | ns | ns |
| *PL <10^th^ perc×Time* | ns | ns | ns |
| *PL >90^th^ perc* | ns | ns | ns |
| *PL >90^th^ perc×Time* | ns | ns | ns |
|  |  |  |  |
| ***CES-D positive affect*** |  |  |  |
| *PL <10^th^ perc* | ns | ***-0.55, p=0.06*** | P<0.10 |
| *PL <10^th^ perc×Time* | ns | ns | ns |
| *PL >90^th^ perc* | ns | **-0.69, p=0.017** | ns |
| *PL >90^th^ perc×Time* | ns | ns | ns |
|  |  |  |  |
| ***CES-D interpersonal problems*** |  |  |  |
| *PL <10^th^ perc* | ns | ns | ns |
| *PL <10^th^ perc×Time* | ns | ns | ns |
| *PL >90^th^ perc* | ns | ns | ns |
| *PL >90^th^ perc×Time* | ns | ns | ns |

Bolded and italicized numbers are for P<0.10 or P<0.05 findings that did not remain significant after correction for multiple testing; Bolded and shaded numbers are for significant findings after correction for multiple testing. “ns” stands for non-significant. TWBCC=Total White Blood Cell Count; PN=Percentage Neutrophils; PL=Percentage Lymphocytes.
